# Risk factors for mortality in preterm infants with necrotizing enterocolitis: a retrospective multicenter analysis

**DOI:** 10.1007/s00431-021-04266-x

**Published:** 2021-10-12

**Authors:** Marcin Kordasz, Michaël Racine, Philipp Szavay, Markus Lehner, Thomas Krebs, Christian Luckert, Eva-Maria Hau, Steffen Berger, Ulf Kessler

**Affiliations:** 1 Department of Pediatric Surgery, Inselspital, Bern University Hospital, University of Bern, 3010 Bern, Switzerland; 2 Department of Visceral Surgery, Inselspital, Bern University Hospital, University of Bern, Bern, Switzerland; 3 Department of Pediatric Surgery, Lucerne Children’s Hospital, Lucerne, Switzerland; 4 Children’s Hospital of Eastern Switzerland, Gallen/Hospital of St, StGallen, St. Gallen, Switzerland; 5 Department of Pediatrics, Inselspital, Bern University Hospital, University of Bern, Bern, Switzerland; 6 Center of Visceral Surgery, Klinik Beau-Site, Hirslanden, Bern, Switzerland; 7 Centre des maladies digestives Lausanne, Clinique Cecil, Hirslanden, Lausanne, Switzerland

**Keywords:** Necrotizing enterocolitis, NEC, Risk factors, Outcome, Mortality

## Abstract

It is difficult to predict the risk of mortality in necrotizing enterocolitis (NEC). This study aimed at identifying risk factors for severe NEC (Bell stage III) and mortality in preterm children with NEC. In this multicenter retrospective study, we analyzed multiple data from 157 premature children with confirmed NEC in the period from January 2007 to October 2018. We performed univariate, multivariate, stepwise logistic regression, and receiver operator characteristics (ROC) analyses. We were able to demonstrate that low Apgar scores (notably at 1′ and 5′), low hemoglobin concentration (Hgb), and high lactate level at disease onset and during disease correlated with NEC severity and mortality (P < 0.05, respectively). Severe NEC was related to congenital heart disease (CHD — OR 2.6, CI95% 1.2–5.8, P 0.015) and patent ductus arteriosus (PDA — OR 3.3, CI95% 1.6–6.9, P 0.0012), whereas death was related to the presence of PDA (OR 5.5, CI95% 2.3–14, P < 0.001).

*Conclusion: *Low Apgar scores, low Hgb, high lactate levels, and the presence of CHD or PDA correlated with severe NEC or mortality in children with NEC.

**Table:** 

**What is Known:** *• It remains difficult to predict which infant that suffers from necrotizing enterocolitis at risk of death.* *• Several clinical and laboratory parameters tools to predict fatal outcome in NEC*.	
**What is New:** *• The following laboratory parameters were associated with the risk of death from NEC: Hemoglobin concentration, base excess and lactate level.* *• The following clinical variables were associated with the risk of death from NEC: Apgar scores, as well as the presence of congenital heart disease and patent ductus arteriosus.*

**Supplementary Information:**

The online version contains supplementary material available at 10.1007/s00431-021-04266-x.

## Introduction

### Background

Necrotizing enterocolitis (NEC) is one of the main causes for morbidity and mortality in preterm neonates [1]. NEC survivors frequently suffer from neurodevelopmental impairment and gastrointestinal sequelae [2, 3]. To date, however, the pathophysiology of NEC, as well as the factors associated with disease severity, has not been sufficiently investigated.

In previous studies, we were able to identify clinical variables (such as such as lactate level, platelet count, birth weight, and Bell stage), which allowed to predict mortality before surgery for NEC [4]. We were also able to show that the mortality rate of NEC patients increased in the presence of CHD or PDA [3, 5], but it is certainly worth noting that the correlation between CHD or PDA and the mortality rate of NEC is not without controversy. Pickard et al. and Bubberman et al., both described that outcomes in terms of mortality were not worse in NEC patients when they had additional CHD [6, 7].

### Objectives

Our previous studies were carried out in monocentric settings, which only included small cohorts. Also, we wanted to include only premature infants since it is not clear whether NEC in term infants is comparable to NEC in preterm infants [8, 9]. In the present study, we have therefore chosen a multicenter retrospective study setting in order to identify the predictive potential of laboratory and clinical parameters, which might have an influence on disease severity and mortality in premature infants with NEC.

## Methods

### Study design

This is a retrospective, multicentric study conducted in Swiss hospitals.

### Setting and participants

We retrospectively collected data from three Swiss Hospitals: The University’s Children Hospital, Bern; the Children’s Hospital, Lucerne; and the Children’s Hospital of Eastern Switzerland, St. Gallen. We identified cases through an X-ray database query, including pre-term patients, with gestational age (GA) below 37 weeks, with confirmed NEC (Bell stage ≥ II [10]) below 4 months of postnatal age in the period from January 2007 to October 2018. We excluded patients with a postnatal age above 120 days or unconfirmed NEC. The data were extracted from medical records, including radiographies.

Our study was approved by the local ethics committee (reference number: 2016–02066).

### Variables

Our primary outcome variables were defined as mortality and disease severity (Bell stage III). We included additional variables, such as birth weight (BW, grams), GA (weeks/days), the number of children per birth, assisted delivery, Apgar (1′, 5′, and 10′), CHD, PDA, congenital malformations or syndromes, age at diagnosis (days), Bell stage, conservative treatment only, NEC surgery, the number of surgical interventions (more than one surgical intervention due to NEC), re-NEC (defined as recurrence of NEC when the child was fully enterally nourished, or the parenteral nutrition stopped), gender, year of birth, and various laboratory parameters before NEC (most recent parameter before disease onset, with a maximum gap of 1 week), at onset (at the time of the earliest therapeutic or diagnostic intervention), and during NEC (worst parameter before the antibiotics were stopped and the child refed). See complete Redcap Data Dictionary Codebook in our supplementary information.

### Data measurement

The data were collected in the RedCap database [11] and analyzed using a combined script written in Perl [12], Python (Python Software Foundation. Python Language Reference, version 2.7. http://www.python.org), and R (R Core Team (2017), R Foundation for Statistical Computing, Vienna, Austria, https://www.R-project.org/).

### Bias

We tried to minimize selection bias by training and coaching local investigators. Above, we performed quality surveys by checking completeness and accuracy of data entry. We used the X-ray and ultrasound database aiming at inclusion of all NEC patients.

### Study size

Study duration but not the number of participants was defined before study onset.

### Quantitative variables

Continuous variables were presented as median and interquartile range (IQR). Discrete variables were approximated to binary variables (yes/no) and presented as the number of positive responses (“yes”) per total number of cases or controls and the percentage of positive responses. When there was no data available for specific variables, we excluded these cases from our analysis. The number of patients having been analyzed for respective variables is marked in the tables in the “*N*” column.

### Statistical methods

We assessed statistical differences between survivors and non-survivors as well as between patients showing Bell stage III and Bell stage II. We used the Mann–Whitney-Wilcoxon test for continuous variables and the chi-square test for discrete variables. To assess the influence of the various parameters on mortality and NEC stage III, we performed univariate and multivariate regression analyses.

For those variables that correlated with mortality or severe NEC, we performed ROC [13] and thereby defined cutoffs for continuous variables. Thereafter, we converted these variables to binary values (defined as yes for continuous values higher than cutoff, and “no” for values lower than cutoff).

Since multiple regression, including stepwise regression, did yield any significant model neither for the prediction of mortality nor for the development of NEC Bell stage III, we performed and reported univariate regression analysis to gain insight about the odds to die or to develop severe disease of the respective variables.

The results were considered statistically significant when the *P* value was less than 0.05.

## Results

### Participants

We included a total of 157 children, of whom 28 (17%) died and 44 (28%) showed NEC stage III.

### Descriptive data

Median GA was 31 weeks 2 days (IQR 6.2). Median BW was 1405 g (IQR 960).

### Outcome data

The significant differences in clinical variables between survivors and non-survivors are given in Table 1.

**Table 1:** Significant differences in patient characteristics between survivors and non-survivors

Variable	*N*	Total	Survivors	Non-survivors	*P*
*n*		157	129	28	
Birth weight (g) (IQR)	157	1405 (960)	1480 (750)	855 (955)	0.0017
Gestational age (weeks/days) (IQR)	157	31.2 (6.2)	32.0 (5.1)	27.0 (5.3)	< 0.001
Assisted delivery *n* (%)	156	133/156, 85%	107/128, 84%	26/28, 93%	0.093
Apgar 5 min (IQR)	153	8 (2)	8 (2)	6 (3)	< 0.001
Age at diagnosis (IQR)	157	9 (12)	8 (11)	11 (13.2)	0.039
Re-NEC *n* (%)	156	8/156, 5%	7/128, 5%	1/28, 4%	0.0025

Overall differences in patient characteristics between survivors and non-survivors, as well as between infants with NEC stage II and stage III, are detailed in Table 1 of our supplementary information.

The significant differences in laboratory parameters between survivors and non-survivors are shown in Table 2.

**Table 2:** Significant differences in laboratory parameters between survivors and non-survivors

Variable	*N*	Total	Survivors	Non-Survivors	*P*
Minimum Hgb (g/l) before NEC onset (IQR)	77	132 (52)	142 (50)	114 (40.2)	0.035
Minimum Hgb (g/l) < 7 days before NEC onset (IQR)	132	144 (53.5)	153 (53)	127 (29)	0.0029
Hgb at disease presentation (g/l) (IQR)	149	140 (50)	146 (49.2)	121 (29)	< 0.001
Minimum Hgb during NEC (g/l) (IQR)	150	114 (35)	119 (37)	103 (19)	0.025
Minimum WBC (G/L) before NEC onset (IQR)	75	7.4 (5.85)	7.71 (5.6)	5.3 (3.78)	0.016
Maximum WBC (G/L) < 7 days before NEC onset (IQR)	120	13.8 (8.93)	13.2 (8.13)	15.6 (10.4)	0.049
Minimum PLT (G/l) before NEC onset (IQR)	74	164 (111)	169 (116)	97 (78)	0.015
Minimum PLT at disease presentation (G/l) (IQR)	148	214 (211)	222 (194)	116 (228)	0.011
Minimum PLT (G/l) during NEC (IQR)	150	157 (172)	174 (168)	57 (133)	0.0024
Minimum pH < 7 days before NEC onset (IQR)	70	7.26 (0.166)	7.26 (0.15)	7.14 (0.157)	0.016
Maximum CRP before NEC onset (IQR)	60	0 (8)	0 (7)	4 (32)	0.1
Maximum CRP (mg/l) < 7 days before NEC onset (IQR)	49	0 (6)	0 (5.25)	0 (24.5)	0.4
CRP at disease presentation (mg/L) (IQR)	135	7 (19.5)	7 (18)	11 (33.5)	0.1
Maximum CRP (mg/l) during NEC (IQR)	141	38 (97)	35 (103)	40.5 (66.5)	0.83
Maximum percentage of premature neutrophils at disease onset (%) (IQR)	109	23 (30)	21 (30.5)	35.5 (30.2)	0.054
Lactate at disease presentation (mmol/L) (IQR)	139	2.2 (2.1)	2.1 (1.8)	3.95 (4.38)	0.0078
Maximum Lactate (mmol/l) during NEC (IQR)	141	2.7 (2.5)	2.4 (2.1)	5.25 (5.85)	< 0.001
Base excess at disease presentation (mmol/L) (IQR)	136	− 2.4 (5.97)	− 2 (5.48)	− 7.9 (6.58)	< 0.001
Maximum base excess during NEC (mmol/L) (IQR)	140	− 3.9 (6.23)	− 3.1 (5.35)	− 10.8 (9.3)	< 0.001
Maximum INR during NEC (IQR)	38	1.34 (0.617)	1.2 (0.6)	1.7 (0.33)	0.024

The entire differences in laboratory parameters between survivors and non-survivors as well as between infants with NEC stage II and III are shown in Table 2 of our supplementary information.

The results of the univariate logistic regression modelling for the risk of development of NEC stage III, as well as for the risk of death, can be seen in our supplementary information, see Tables 3, 4,5 and 7. The predictors in favor of mortality, including cutoffs on the basis of the ROC analysis, are shown in Table 3. Predictors of severe NEC are displayed in Table 8 in our supplementary information. The types of CHD are given in table 10 in our supplementary information.

**Table 3:** Predictors of mortality, univariate regression (*NA* not applicable)

	**ROC analysis**						**Univariate regression**		
Value	AUC	cutoff	Sensitivity	Specificity	PPV	NPV	OR	CI95%	*P*
Bell stage III	NA	NA	NA	NA	NA	NA	7.1	3–18	< 0.001
Gestational age (weeks/days)	0.7	29.0	0.71	0.7	0.34	0.92	5.8	2.4–15	< 0.001
Apgar 1 min	0.73	5.5	0.75	0.57	0.28	0.91	4	1.7–11	0.0033
Apgar 5 min	0.7	6.5	0.57	0.8	0.39	0.89	4.8	2.1–12	< 0.001
Apgar 10 min	0.7	7.5	0.61	0.78	0.39	0.9	3.2	1.3–8.2	0.011
PDA	NA	NA	NA	NA	NA	NA	5.5	2.3–14	< 0.001
Conservative treatment only	NA	NA	NA	NA	NA	NA	0.4	0.17–0.93	0.032
NEC surgery	NA	NA	NA	NA	NA	NA	2.5	1.1–5.8	0.032
Minimum Hb (g/l) < 7 days before NEC onset	0.7	141	0.78	0.6	0.29	0.93	3.9	1.6–10	0.0042
Minimum Hb during NEC (g/l)	0.64	113	0.76	0.56	0.26	0.92	3.1	1.2–8.1	0.018
Minimum pH < 7 days before NEC onset	0.75	7.2	0.67	0.84	0.38	0.94	10	2.3–56	0.0032
Hgb at disease presentation (g/l)	0.72	141	0.88	0.56	0.29	0.96	3.6	1.5–9.4	0.0061
Lactate at disease presentation (mmol/L)	0.68	3.8	0.55	0.83	0.38	0.91	3.8	1.4–10	0.0079
Maximum Lactate (mmol/l) during NEC	0.78	3.8	0.77	0.75	0.36	0.95	3.4	1.3–8.8	0.011

The results of the multivariate logistic regression are presented in our supplementary information — table 6 for the risk of severe NEC and table 9 for mortality, respectively. As stated above, no significant results were yielded from this analysis.

### Main results

The values for BW, GA, Apgar values at 1, 5, and 10 min, as well as frequency of assisted delivery, were significantly different between survivors and non-survivors (*P* < 0.05, respectively, see Table 1, and Table 1 in our supplementary information).

Apgar scores below 6 at 1 min (OR 4, 1.7–11, *P* 0.0033), under 7 at 5 min (OR 4.8, 2.1–12, *P* < 0.001) and under 8 at 10 min (OR 3.2, 1.3–8.2, P 0.011) as well as NEC surgery (OR 2.5, CI95% 1.1–5.8, *P* 0.032) correlated with an increased death rate (Table 3).

Furthermore, severe NEC correlated with mortality (OR 7.1, CI95% 3–18, *P* < 0.001), just like the presence of PDA (OR 5.5, CI95% 2.3–14, *P* < 0.001).

The following laboratory parameters were associated with an increased mortality rate: before disease onset: Hgb lower than 141 g/l (OR 3.9, 1.6–10, *P* 0.0042), minimum pH lower than 7.2 (OR 10, 2.3–56, *P* 0.0032); at disease onset: Hgb lower than 141 g/l (OR 3.6, 1.5–9.4, *P* 0.0061) and lactate over 3.8 mmol/l (OR 3.8, 1.4–10, *P* 0.0079); during the course of disease: Hgb below 113 g/l (OR 3.1, 1.2–8.1, P 0.018) and lactate over 3.8 mmol/l (OR 3.4, 1.3–8.8, *P* 0.011) (Table 3).

NEC stage III occurred more often in infants with a GA under 31 weeks and 2 days (OR 4.5, 2.1–10, *P* < 0.001). Lower Apgar scores (less than 6 at 1 min, OR 2.4, 1.2–5.1, *P* 0.019 and less than 8 at 5 min, OR 3.2, 1.5–7, *P* 0.0033) could be related to severe NEC (table 7 of our supplementary information). In addition, the presence of CHD (OR 2.6, CI95% 1.2–5.8, *P* 0.015) and PDA (OR 3.3, CI95% 1.6–6.9, *P* 0.0012) increased the risk of severe NEC.

Finally, surgical treatment correlated significantly with the development of severe NEC (OR 61, CI95% 21–223, *P* < 0.001).

The laboratory parameters at disease onset that corresponded with NEC stage III included a proportion of immature neutrophils above 34% (OR 2.9, 1.2–7.4, P 0.025) and a lactate level over 2.6 mmol/l (OR 3.7, 1.7–8.3, *P* 0.0012). During the disease, severe NEC correlated with a WBC higher than 22G/l (OR 4, 1.8–9.3, *P* < 0.001) and a lactate level above 3.8 mmol/l (OR 4.2, 1.9–9.7, *P* < 0.001).

## Discussion

### Key results

The primary aim of this study was to gain insight into the factors influencing disease severity and mortality in premature children with NEC.

We were able to show that the presence of PDA tripled the risk of severe NEC, thus increasing the risk of death by a factor of five. CHD also tripled the risk of severe NEC.

Above that low Apgar scores, low Hgb, and high lactate levels correlated with severe NEC and mortality.

### Limitations

Due to the retrospective study design, our study bears several limitations.

Patient treatment was at the discretion of the attending physician. We have found some minor differences of the clinical protocols concerning NEC relevant features like postnatal feeding, PDA management, or NEC treatment between the participating centers. However, due to copyright issues, we were not able to provide these protocols.

Heterogeneity of patient management also led to missing data, e.g., for laboratory parameters, which might thereby be biased.

Also, timing of the laboratory investigations was set at a relatively wide range of time and not reported in detail.

Using an X-ray and ultrasound database to identify NEC patients might have caused incomplete patient inclusion, such as patients with fulminant NEC.

In order to control such methodological problems in future studies, the differences in the NEC-relevant clinical protocols between the participating centers should be precisely documented, as we consider it very difficult to control the important NEC-relevant influencing factors in a uniform protocol for the management of premature infants.

Although we aimed to include a large number of patients by using a multicenter approach, we were not able to gain significant results by means of multiple regression analysis. We believe that statistically more robust knowledge about predictive factors for NEC could only be obtained from studies in larger patient populations.

### Interpretation

These results confirm our previous finding that PDA seems to be a risk factors for mortality [5]. Furthermore, as suggested by Carlo et al. [14], our results support the pathophysiological theory, according to which infants with PDA or CHD are at risk for NEC due to local intestinal ischemia. In contrast to our own previous findings, CHD was not correlated with mortality. This is in accordance with the findings by others [6, 7] and warrants, as discussed above, evaluation in a larger cohort.

Our study supports other previous findings [15–18], according to which low BW and GA must be seen as risk factors for the development of severe NEC (Bell stage III).

Low Apgar scores (below 6 at 1 min and below 8 at 5 min) could be related to more severe disease courses, which is consistent with findings of Stafford et al. [19], who were able to show that lower Apgar scores at 1 and 5 min can be associated with severe NEC.

It has already been shown that hyperlactatemia serves as predictor for poor outcomes in the treatment of critically ill children [20]. In 2003, Abubacker et al. demonstrated that pre-operative hyperlactatemia can possibly serve as predictor in babies with NEC [21]. Our previous study confirmed this assumption [4]. As the results of this study confirm, lactate values are clearly related to severe NEC, both at disease presentation and during progression of NEC.

To the best of our knowledge, our study is the first to show that there is a significant association between lactate levels, disease severity, and mortality at disease onset, which, hopefully, will allow for a better and faster treatment of NEC in the future. The fact that we could extract data from a large multicenter patient group of preterm infants can be seen as strength of our study. We believe that our patient selection criteria as well as our data extraction methods reduced selection and observer bias.

### Generalizability

Our study supports the current knowledge, that some influencing factors (such as low Apgar scores, congenital malformations, low Hgb, elevated lactate levels, and the presence of CHD or PDA,) might be helpful to predict the severity of NEC, and to adjust treatment measures for better disease outcomes. However, further studies, including prospective trials, are needed to confirm these variables as risk factors.

## Supplementary Information

Below is the link to the electronic supplementary material.

Supplementary file1 (PDF 507 KB)

Supplementary file2 (PDF 63 KB)

## Data Availability

Data has been submitted as supplementary material.

## Abbreviations

BW: birth weight
CHD: congenital heart disease
CI95: 95% confidence interval
GA: gestational age
Hgb: hemoglobin concentration
INR: international normalized ratio
NA: not applicable
NEC: necrotizing enterocolitis
PDA: patent ductus arteriosus
PLT: platelet count
ROC: receiver operator characteristics
WBC: white blood cell count

## Code availability

N/A.
