# Supplementary material for: Risk factors for mortality in preterm infants with necrotizing enterocolitis: a retrospective multicenter analysis
Source: Eur J Pediatr. 2021 Oct 12;181(3):933–9. doi: 10.1007/s00431-021-04266-x (PMC8897343; doi:10.1007/s00431-021-04266-x)
Supplement: Supplementary file 1 — Supplementary file1 (PDF 507 KB) [file 431_2021_4266_MOESM1_ESM.pdf]

Variable / Field Name	Field Label *Field Note*	Field Attributes (Field Type, Validation, Choices, Calculations, etc.)	
1	record_id	Record ID	text
2	birth_weight	Section Header: *Perinatal period*<br>Birth weight (in g)<br>*number; in gramm; e.g. 1560*	text (number, Min: 450, Max: 9999)
3	birth_length	Birth length (in cm)<br>*number; in cm, e.g. 40*	text (number, Min: 20, Max: 65)
4	birth_head	Birth head circumference<br>*integer, in cm, e.g. 35*	text (integer, Min: 17, Max: 50)
5	ga_weeks	Gestational age (weeks. days)<br>*number, 2 digits and 1 decimal, weeks.days, e.g. 26.2*	text (number_1dp, Min: 22, Max: 41)
6	other_comments_pregna2	Comments pregnancy<br>*free text*	notes
7	no_infants	Number of infants in this birth	dropdown<br>1 | 1<br>2 | 2<br>3 | 3<br>4 | 4<br>5 | 5<br>99 | unknown
8	delivery	Delivery mode	dropdown<br>1 | spontaneous<br>2 | assisted vaginal delivery<br>3 | cesarian section<br>99 | unknown

9	apgar_1	Apgar 1 minute<br>*number, 0-10; e.g. 9*	text (integer, Min: 0, Max: 10)
10	apgar_5	Apgar 5 minutes<br>*number, 0-10; e.g. 9*	text (integer, Min: 0, Max: 10)
11	apgar_10	Apgar 10 minutes<br>*number, 0-10; e.g. 9*	text (integer, Min: 0, Max: 10)
12	del_temperature	Temperature at delivery/admission<br>*in Celcius degrees, for example 37.1*	text (number_1dp)
13	chd	Congenital heart disease	dropdown<br>0 | no<br>3 | yes<br>99 | unknown
14	other_chd<br>Show the field ONLY if:<br>[chd] = '3' or [chd] = '2'	Other congenital heart disease: description<br>*free text*	notes
15	pda_treated	PDA	dropdown<br>1 | none<br>2 | treated medically<br>3 | treated surgically
16	chd_cat	Congenital heart disease categories (nach Pavlovic)	dropdown (autocomplete)<br>1 | diseases with relevant reverse abdominal flow (A)<br>2 | diseases with lower systolic output (B)<br>3 | cyanotic heart diseases (C)<br>4 | diseases with shunts (D)

			5 pulmonary hypertension (E)
17	malformations	Congenital malformations or syndroms	dropdown<br>0 no<br>1 minor (1 major organ affected)<br>2 major (more than one major organ affected)<br>3 syndromes<br>99 unknown
18	chromosomal_malformations	Chromosomal malformations	yesno<br>1 Yes<br>0 No
19	malformations_desc<br>Show the field ONLY if:<br>[malformations] = '1' or [malformations] = '2'	Congenital malformations or syndromes: description<br>*free text*	notes
20	spont_perforation	Section Header: *NEC parameters*<br>Focal gastrointestinal perforation/spontaneous gastrointestinal perforation (separate from NEC!)	dropdown<br>0 no<br>1 yes<br>99 unknown
21	init_symptoms_date	Initial symptoms date<br>*dd.mm.yyyy*	text (date_dmy)
22	dx_date	Date at Diagnosis (start non per os including gut priming)	text (date_dmy, Min: 1970-01-01, Max: 2050-12-31)

23	age_at_diagnosis	Age at diagnoses (calculated)<br>*calculated field; no data entry*	calc<br>Calculation:<br>datediff([patient_dob],[dx_date],"d","dmy")
24	bell	Bell staging (max documented grade)- always report worst documented grading during disease	dropdown, Required<br>21 stage 2a<br>22 stage 2b<br>31 stage 3a<br>32 stage 3b<br>88 not applicable<br>99 unknown
25	severity	Disease severity<br>*no need to fill in, calculated automatically*	dropdown<br>1 stad 1 bell not deceased within 48h<br>2 stad 2 bell not deceased within 48h<br>3 stad 3 bell not deceased within 48h<br>4 deceased during first 48 h of NEC<br>88 not applicable<br>99 unknown
26	conservative_treatment	Section Header: *NEC treatment*<br>Conservative treatment only	dropdown<br>0 no<br>1 yes<br>99 unknown
27	treat_comment	Comments conservative treatment<br>*free text*	notes
28	nec_surgery	NEC/FIP surgery	dropdown<br>0 no

			1 yes<br>99 unknown
29	no_surgeries	Number of surgeries due to NEC/FIP	dropdown<br>0 no<br>1 1x<br>2 2x<br>3 >2x<br>99 unknown
30	date_necsurg1<br>Show the field ONLY if:<br>[no_surgeries] = '1' or [no_surgeries] = '2' or [no_surgeries] = '3' or [no_surgeries] = '9'	Date of 1st NEC surgery	text (date_dmy)
31	surg1_approach<br>Show the field ONLY if:<br>[no_surgeries] = '1' or [no_surgeries] = '2' or [no_surgeries] = '3' or [no_surgeries] = '9'	1st NEC surgery: surgical approach	dropdown<br>1 only drainage<br>2 laparotomy without intestinal incision/resection<br>3 Laparotomy with resection and stoma<br>4 Laparotomy with resection and anastomosis<br>5 Laparotomy with resection and anastomosis and stoma<br>6 other<br>88 not applicable

			99 | unknown
32	surg1_desc_appr<br>Show the field ONLY if:<br>[no_surgeries] = '1' or [no_surgeries] = '2' or [no_surgeries] = '3' or [no_surgeries] = '9'	1st NEC surgery: description of surgical approach<br>*free text*	notes
33	surg1_reason<br>Show the field ONLY if:<br>[no_surgeries] = '1' or [no_surgeries] = '2' or [no_surgeries] = '3' or [no_surgeries] = '9'	Reason for 1st surgery	checkbox<br>1 | surg1_reason___1 | fast-progression<br>2 | surg1_reason___2 | passage problem/ obstruction<br>3 | surg1_reason___3 | perforation<br>4 | surg1_reason___4 | Other<br>5 | surg1_reason___5 | complication<br>99 | surg1_reason___99 | unknown
34	surg1_reason_other<br>Show the field ONLY if:<br>[no_surgeries] = '2' or [no_surgeries] = '3' or [no_surgeries] = '1' or [no_surgeries] = '9'	Other reason for 1st surgery<br>*free text*	notes
35	date_necsurg2<br>Show the field ONLY if:	Date of 2nd NEC surgery	text (date_dmy)

	[no_surgeries] = '2' or [no_surgeries] = '3' or [no_surgeries] = '9'		
36	surg2_approach<br>Show the field ONLY if:<br>[no_surgeries] = '2' or [no_surgeries] = '3' or [no_surgeries] = '9'	2nd NEC surgery: surgical approach	dropdown<br>1 — only drainage<br>2 — laparotomy without intestinal incision/resection<br>3 — Laparotomy with resection and stoma<br>4 — Laparotomy with resection and anastomosis<br>5 — other<br>88 — not applicable<br>99 — unknown
37	surg2_desc_appr<br>Show the field ONLY if:<br>[no_surgeries] = '2' or [no_surgeries] = '3' or [no_surgeries] = '9'	2nd NEC surgery: description of other surgical approach<br>*free text*	notes
38	surg2_reason<br>Show the field ONLY if:<br>[no_surgeries] = '2' or [no_surgeries] = '3' or [no_surgeries] = '9'	Reason for 2nd surgery	checkbox<br>1 — surg2_reason___1 — re-NEC<br>2 — surg2_reason___2 — second look<br>3 — surg2_reason___3 — complication<br>4 — surg2_reason___4 — Other<br>5 — surg2_reason___5 — Stoma closure<br>99 — surg2_reason___99 — unknown

39	surg2_reason_desc<br>Show the field ONLY if:<br>[no_surgeries] = '2' or [no_surgeries] = '3' or [no_surgeries] = '9'	Reason for 2nd surgery (details, other...)<br>*free text*	notes
40	date_necsurg3<br>Show the field ONLY if:<br>[no_surgeries] = '3' or [no_surgeries] = '9'	Date of 3rd NEC surgery	text (date_dmy)
41	surg3_approach<br>Show the field ONLY if:<br>[no_surgeries] = '3' or [no_surgeries] = '9'	3rd NEC surgery: surgical approach	dropdown<br><table><tr><td>1</td><td>only drainage</td></tr><tr><td>2</td><td>laparotomy without intestinal incision/resection</td></tr><tr><td>3</td><td>Laparotomy with resection and stoma</td></tr><tr><td>4</td><td>Laparotomy with resection and anastomosis</td></tr><tr><td>5</td><td>other</td></tr><tr><td>88</td><td>not applicable</td></tr><tr><td>99</td><td>unknown</td></tr></table>
42	surg3_desc_appr<br>Show the field ONLY if:<br>[no_surgeries] = '3' or [no_surgeries] = '9'	3rd NEC surgery: description of surgical approach<br>*free text*	notes
43	surg3_reason	Reason for 3rd surgery	checkbox

	Show the field ONLY if:<br>[no_surgeries] = '3' or [no_surgeries] = ' 9'		<table><tr><td>1</td><td>surg3_reason___1</td><td>re-NEC</td></tr><tr><td>2</td><td>surg3_reason___2</td><td>second look</td></tr><tr><td>3</td><td>surg3_reason___3</td><td>complication</td></tr><tr><td>4</td><td>surg3_reason___4</td><td>Other</td></tr><tr><td>5</td><td>surg3_reason___5</td><td>Stoma closure</td></tr><tr><td>99</td><td>surg3_reason___99</td><td>unknown</td></tr></table>
44	surg3_reason_desc<br>Show the field ONLY if:<br>[no_surgeries] = '3' or [no_surgeries] = ' 9'	Reason for 3rd surgery (details, other...)<br>*free text*	notes
45	re_nec_1	Section Header: *Re-NEC*<br>RE-NEC or Re-FIP (defined as recurrence of NEC when child is fully enteral nourished/ PEN stopped)	dropdown<table><tr><td>0</td><td>no</td></tr><tr><td>1</td><td>yes</td></tr><tr><td>99</td><td>not documented</td></tr></table>
46	re_nec_com<br>Show the field ONLY if:<br>[re_nec_1] = '1'	Re-NEC comment (date, bell stage and localization)<br>*free text*	notes
47	spont_perforation2<br>Show the field ONLY if:<br>[re_nec_1] = '1'	Re-NEC: Focal gastrointestinal perforation/spontaneous gastrointestinal perforation (separate from NEC!)	dropdown<table><tr><td>0</td><td>no</td></tr><tr><td>1</td><td>yes</td></tr><tr><td>99</td><td>unknown</td></tr></table>
48	init_symptoms_date2	Initial symptoms date of 2nd NEC<br>*dd.mm.yyyy*	text (date_dmy)

	Show the field ONLY if:<br>[re_nec_1] = '1'		
49	dx_date2<br>Show the field ONLY if:<br>[re_nec_1] = '1'	2nd NEC: Date at Diagnosis (start NPO)	text (date_dmy, Min: 01.01.1970, Max: 31.12.2050)
50	age_at_diagnosis2<br>Show the field ONLY if:<br>[re_nec_1] = '1'	2nd NEC: Age at diagnoses (calculated)<br>*calculated field; no data entry*	calc<br>Calculation:<br>datediff([patient_dob],[dx_date2],"d","dmy")
51	bell2<br>Show the field ONLY if:<br>[re_nec_1] = '1'	2nd NEC: Bell staging (max grade)- always report worst grading during disease	dropdown, Required<br>1 stad 1<br>21 stad 2a<br>22 stad 2b<br>31 stad 3a<br>32 stad 3b<br>88 not applicable<br>99 unknown
52	conserv2<br>Show the field ONLY if:<br>[re_nec_1] = '1'	2nd NEC: Conservative treatment only	dropdown (autocomplete)<br>0 no<br>1 yes<br>99 unknown
53	pen_stop_renec	PEN stopped on	text (date_dmy)
54	complications_core	Section Header: *Outcomes*<br>Complications<br>*free text*	notes

55	survival	Survival: last known status	dropdown<br>0 | no<br>1 | yes<br>99 | unknown
56	date_knownalive	Last date known alive (date of last report)	text (date_dmy)
57	died_duetonec<br>Show the field ONLY if:<br>[survival] = '0'	Died due to NEC	dropdown<br>0 | no<br>1 | yes<br>2 | unclear<br>99 | unknown
58	exit_cause_desc<br>Show the field ONLY if:<br>[survival] = '0'	Death cause description<br>*free text*	notes
59	exitus_date<br>Show the field ONLY if:<br>[survival] = '0'	Exitus date	text (date_dmy)
60	discharge_date_hosp	Date of first discharge to other hospital	text (date_dmy)
61	discharge_home	Date of discharge home	text (date_dmy)
62	hosp_duration	Hospitalisation duration<br>*no need to input - calculated externally!*	text (integer)<br>Field Annotation: From birth to first discharge or if re-NEC, duration of the last hospitalisation
63	total_hosp_duration	Total hospitalisation duration<br>*no need to input - calculated externally!*	text (integer)

64	core_data_complete	Section Header: *Form Status*<br>Complete?	dropdown<br>0 Incomplete<br>1 Unverified<br>2 Complete
