# Supplementary material for: Risk factors for mortality in preterm infants with necrotizing enterocolitis: a retrospective multicenter analysis
Source: Eur J Pediatr. 2021 Oct 12;181(3):933–9. doi: 10.1007/s00431-021-04266-x (PMC8897343; doi:10.1007/s00431-021-04266-x)
Supplement: Supplementary file 2 — Supplementary file2 (PDF 63 KB) [file 431_2021_4266_MOESM2_ESM.pdf]

Supplementary Tables

Table I: Clinical parameters

Variable	N	Total	Survivors	Non-Survivors	P	Bell III	Bell II	P
N	157		129	28		44	113	
Birth weight [g] (IQR)	157	1405 (960)	1480 (750)	855 (955)	0.0017	1045 (894)	1500 (790)	0.0022
Birth weight percentile (IQR)	157	27 (41)	25 (43)	32 (24)	0.8	29.5 (36.2)	26 (41)	0.85
Birth length [cm] (IQR)	150	40.5 (9)	41 (7)	33.2 (6.75)	<0.001	36.8 (8.62)	41 (7)	0.006
Birth length percentile (IQR)	150	37 (41.8)	36.5 (45.5)	37.5 (30.8)	0.55	37 (31.2)	37 (46.2)	0.73
Birth head circumference [cm] (IQR)	127	28 (6)	29 (5)	24 (3.5)	<0.001	25 (4.5)	29 (5.25)	0.0029
Birth head circumference percentile (IQR)	128	31 (37)	31.5 (41.2)	28 (36.8)	0.47	26 (33.5)	32 (42)	0.16
Gestational age [weeks.days] (IQR)	157	31.2 (6.2)	32.0 (5.1)	27.0 (5.3)	<0.001	28.2 (5.2)	32.0 (5.3)	0.0011
Number of infants in this birth (IQR)	157	1 (1)	1 (1)	1 (1)	0.73	1 (1)	1 (1)	0.089
Assisted delivery n (%)	156	133/156, 85%	107/128, 84%	26/28, 93%	0.093	38/44, 86%	95/112, 85%	0.045
Apgar 1 minute (IQR)	154	6 (3)	6 (3)	3 (3)	<0.001	5 (4)	6 (3)	0.0064
Apgar 5 minutes (IQR)	153	8 (2)	8 (2)	6 (3)	<0.001	7 (2)	8 (2)	0.0043
Apgar 10 minutes (IQR)	151	9 (2)	9 (1)	7 (3)	<0.001	8 (2)	9 (1)	0.0067
Congenital heart disease n (%)	156	36/156, 23%	26/128, 20%	10/28, 36%	0.71	16/44, 36%	20/112, 18%	0.56
PDA n (%)	156	60/156, 38%	40/128, 31%	20/28, 71%	0.75	26/44, 59%	34/112, 30%	0.88
Congenital malformations or syndroms n (%)	152	33/152, 22%	27/124, 22%	6/28, 21%	0.41	9/44, 20%	24/108, 22%	0.24
Age at diagnosis (IQR)	157	9 (12)	8 (11)	11 (13.2)	0.039	9 (9.25)	8 (14)	0.87
Conservative treatment only n (%)	157	101/157, 64%	88/129, 68%	13/28, 46%	1	4/44, 9%	97/113, 86%	0.017
NEC surgery n (%)	157	56/157, 36%	41/129, 32%	15/28, 54%	1	40/44, 91%	16/113, 14%	0.017
More than one surgery due to NEC n (%)	157	40/157, 25%	34/129, 26%	6/28, 21%	0.52	28/44, 64%	12/113, 11%	0.43
Male gender n (%)	157	85/157, 54%	71/129, 55%	14/28, 50%	1	26/44, 59%	59/113, 52%	1
Year of birth (IQR)	157	2011 (2007-2018)	2011 (2007-2018)	2012 (2007-2017)	0.52	2012 (2007-2018)	2011 (2007-2017)	0.19
Re-NEC n (%)	156	8/156, 5%	7/128, 5%	1/28, 4%	0.0025	1/44, 2%	7/112, 6%	<0.001

Table II: Laboratory parameters

Variable	N	Total	Survivors	Non-Survivors	P	Bell III	Bell II	P
Umbilical artery pH (IQR)	128	7.3 (0.09)	7.3 (0.0825)	7.32 (0.11)	0.37	7.32 (0.07)	7.3 (0.09)	0.5
Umbilical venous pH (IQR)	38	7.35 (0.0925)	7.36 (0.076)	7.34 (0.115)	0.31	7.35 (0.13)	7.35 (0.118)	0.45
Minimum Hb [g/l] before NEC onset (IQR)	77	132 (52)	142 (50)	114 (40.2)	0.035	120 (61)	137 (49.5)	0.092
Maximum Hb [g/l] before NEC onset (IQR)	77	183 (32)	184 (33)	176 (15)	0.063	178 (36)	184 (30.5)	0.62
Minimum Hb [g/l] <7 days before NEC onset (IQR)	132	144 (53.5)	153 (53)	127 (29)	0.0029	140 (48)	148 (55.5)	0.28
Minimum Hb [g/l] 1-2 weeks before NEC onset (IQR)	76	145 (44.2)	144 (45.5)	147 (46)	0.82	143 (44.8)	145 (44.2)	0.7
Hb at disease presentation [g/l] (IQR)	149	140 (50)	146 (49.2)	121 (29)	<0.001	126 (45)	144 (48)	0.017
Minimum Hb during NEC [g/l] (IQR)	150	114 (35)	119 (37)	103 (19)	0.025	102 (34)	122 (36)	<0.001
Minimum WBC [G/L] before NEC onset (IQR)	75	7.4 (5.85)	7.71 (5.6)	5.3 (3.78)	0.016	7 (4.51)	7.42 (5.65)	0.77
Maximum WBC [G/L] before NEC onset (IQR)	75	15.3 (10.2)	15.3 (11)	15.3 (3.05)	0.95	14.3 (9.8)	15.7 (9.47)	0.71
Minimum WBC [G/L] <7 days before NEC onset (IQR)	120	9.53 (6.71)	9.4 (6.61)	10.9 (6.06)	0.81	9.49 (7.38)	9.54 (6.45)	0.86
Maximum WBC [G/L] <7 days before NEC onset (IQR)	120	13.8 (8.93)	13.2 (8.13)	15.6 (10.4)	0.049	14.7 (11.2)	13.1 (8.03)	0.19
WBC at disease presentation [G/l] (IQR)	149	9.53 (7.8)	9.48 (6.57)	11.6 (11.9)	0.43	9.98 (14)	9.53 (6.74)	0.74
Maximum WBC [G/l] during NEC (IQR)	149	17 (13.2)	16.4 (13)	22 (16.7)	0.69	24.2 (14.4)	14.8 (11.2)	0.0011
Minimum PLT [G/l] before NEC onset (IQR)	74	164 (111)	169 (116)	97 (78)	0.015	146 (82)	169 (122)	0.22
Minimum PLT [G/l] <7 days before NEC onset (IQR)	120	198 (142)	210 (129)	178 (226)	0.19	174 (102)	223 (133)	0.057
Minimum PLT at disease presentation [G/l] (IQR)	148	214 (211)	222 (194)	116 (228)	0.011	156 (124)	255 (203)	<0.001
Minimum PLT [G/l] during NEC (IQR)	150	157 (172)	174 (168)	57 (133)	0.0024	49 (79)	193 (177)	<0.001
Maximum total bilirubin <7 days before NEC onset [mcmol/l] (IQR)	122	151 (75.5)	155 (74.5)	123 (58)	0.071	154 (70.2)	148 (74.8)	0.59
Maximal lactate [mmol/l] before NEC onset (IQR)	74	3 (2.27)	2.9 (1.9)	5.2 (4.3)	0.069	3.25 (2.62)	2.9 (1.95)	0.11
Maximal lactate [mmol/l] <7 days before NEC onset (IQR)	72	2.5 (1.32)	2.4 (1.2)	2.8 (2.8)	0.17	2.75 (0.65)	2.4 (1.25)	0.056
Lactate at disease presentation [mmol/L] (IQR)	139	2.2 (2.1)	2.1 (1.8)	3.95 (4.38)	0.0078	4.05 (3.65)	1.9 (1.3)	<0.001
Maximum Lactate [mmol/l] during NEC (IQR)	141	2.7 (2.5)	2.4 (2.1)	5.25 (5.85)	<0.001	5.25 (4.05)	2.3 (1.8)	<0.001
Minimum pH before NEC onset (IQR)	72	7.21 (0.118)	7.21 (0.121)	7.19 (0.101)	0.17	7.19 (0.161)	7.21 (0.112)	0.84
Minimum pH <7 days before NEC onset (IQR)	70	7.26 (0.166)	7.26 (0.15)	7.14 (0.157)	0.016	7.21 (0.198)	7.26 (0.159)	0.1
Maximum CRP before NEC onset (IQR)	60	0 (8)	0 (7)	4 (32)	0.1	4 (9)	0 (7)	0.27
Maximum CRP [mg/l] <7 days before NEC onset (IQR)	49	0 (6)	0 (5.25)	0 (24.5)	0.4	0 (8.5)	0 (3)	0.49
CRP at disease presentation [mg/L] (IQR)	135	7 (19.5)	7 (18)	11 (33.5)	0.1	11 (26)	6.5 (18.2)	0.11
Maximum CRP [mg/l] during NEC (IQR)	141	38 (97)	35 (103)	40.5 (66.5)	0.83	98 (126)	29.5 (71)	<0.001
Procalcitonin at disease presentation (IQR)	1	0.65 (0)	0.65 (0)	NA (NA)	1	NA (NA)	0.65 (0)	1
Maximum Procalcitonin during NEC [mcg/l] (IQR)	2	0.78 (0.13)	0.78 (0.13)	NA (NA)	1	NA (NA)	0.78 (0.13)	1
Percentage of premature WBC at disease onset [%] = I/T ratio (IQR)	2	16.5 (0.5)	16.5 (0.5)	NA (NA)	1	NA (NA)	16.5 (0.5)	1
Maximum percentage of premature neutrophils at disease onset [%] (IQR)	109	23 (30)	21 (30.5)	35.5 (30.2)	0.054	38 (28.2)	19 (28)	<0.001
Maximum percentage of premature WBC at disease onset [%] (IQR)	103	18 (21.5)	18 (20)	23 (23)	0.42	24 (19)	16 (20)	0.026
Base excess at disease presentation [mmol/L] (IQR)	136	-2.4 (5.97)	-2 (5.48)	-7.9 (6.58)	<0.001	-6.4 (5.1)	-1.8 (5.3)	<0.001
Maximum base excess during NEC [mmol/L] (IQR)	140	-3.9 (6.23)	-3.1 (5.35)	-10.8 (9.3)	<0.001	-8.9 (6.25)	-2.9 (5)	<0.001
Maximum PTT after disease onset (IQR)	21	48.4 (25)	47.3 (22.1)	72 (35)	0.076	53.5 (22.5)	46.2 (23.2)	0.46
Maximum INR during NEC (IQR)	38	1.34 (0.617)	1.2 (0.6)	1.7 (0.33)	0.024	1.7 (0.55)	1.17 (0.34)	0.0022

Table III: Univariate regression - risk of death

Value	OR	CI95%	P
Bell III	7.1	3 - 18	<0.001
Birth weight [g]	1	1 - 1	0.0019
Birth weight percentile	1	0.98 - 1	0.93
Birth length [cm]	0.83	0.75 - 0.91	<0.001
Birth length percentile	0.99	0.97 - 1	0.42
Birth head circumference [cm]	0.78	0.67 - 0.9	<0.001
Birth head circumference percentile	0.99	0.97 - 1	0.44
Gestational age [weeks.days]	0.97	0.95 - 0.99	<0.001
Number of infants in this birth	1.2	0.61 - 2.3	0.56
Assisted delivery	2.6	0.69 - 17	0.22
Apgar 1 minute	0.68	0.55 - 0.82	<0.001
Apgar 5 minutes	0.68	0.54 - 0.84	<0.001
Apgar 10 minutes	0.65	0.49 - 0.83	<0.001
Congenital heart disease	2.2	0.88 - 5.2	0.084
PDA	5.5	2.3 - 14	<0.001
Congenital malformations or syndroms	0.98	0.33 - 2.5	0.97
Age at diagnosis	1	1 - 1.1	0.013
Conservative treatment only	0.4	0.17 - 0.93	0.032
NEC surgery	2.5	1.1 - 5.8	0.032
More than one surgery due to NEC	0.76	0.26 - 1.9	0.59
Male gender	0.82	0.36 - 1.9	0.63
Year of birth	1	0.91 - 1.2	0.6
Re-NEC	0.64	0.034 - 3.8	0.68
Umbilical artery pH	6.5	0.034 - 2646	0.51
Umbilical venous pH	0.11	0 - 133	0.51

Table IV: Univariate regression - risk of death

Value	OR	CI95%	P
Minimum Hb [g/l] before NEC onset	0.98	0.96 - 1	0.049
Maximum Hb [g/l] before NEC onset	0.99	0.97 - 1	0.34
Minimum Hb [g/l] <7 days before NEC onset	0.98	0.97 - 0.99	0.0065
Minimum Hb [g/l] 1-2 weeks before NEC onset	1	0.98 - 1	0.88
Hb at disease presentation [g/l]	0.97	0.95 - 0.99	<0.001
Minimum Hb during NEC [g/l]	0.98	0.96 - 0.99	0.019
Minimum WBC [G/L] before NEC onset	0.78	0.58 - 0.96	0.049
Maximum WBC [G/L] before NEC onset	0.97	0.89 - 1	0.49
Minimum WBC [G/L] <7 days before NEC onset	1	0.94 - 1.1	0.86
Maximum WBC [G/L] <7 days before NEC onset	1	0.99 - 1.1	0.13
WBC at disease presentation [G/l]	1	0.97 - 1.1	0.4
Maximum WBC [G/l] during NEC	1	0.99 - 1.1	0.13
Minimum PLT [G/l] before NEC onset	0.99	0.97 - 1	0.019
Minimum PLT [G/l] <7 days before NEC onset	1	1 - 1	0.92
Minimum PLT at disease presentation [G/l]	1	0.99 - 1	0.037
Minimum PLT [G/l] during NEC	0.99	0.99 - 1	0.018
Maximum total bilirubin <7 days before NEC onset [mcmol/l]	0.99	0.99 - 1	0.13
Maximal lactate [mmol/l] before NEC onset	1.6	1.2 - 2.4	0.0072
Maximal lactate [mmol/l] <7 days before NEC onset	1.6	1 - 2.5	0.045
Lactate at disease presentation [mmol/L]	1.3	1.1 - 1.6	0.0012
Maximum Lactate [mmol/l] during NEC	1.4	1.2 - 1.6	<0.001
Minimum pH before NEC onset	0.0072	0 - 13	0.2
Minimum pH <7 days before NEC onset	1e-04	0 - 0.18	0.021
Maximum CRP before NEC onset	1	1 - 1.1	0.03
Maximum CRP [mg/l] <7 days before NEC onset	1	1 - 1.1	0.082
CRP at disease presentation [mg/L]	1	0.99 - 1	0.13
Maximum CRP [mg/l] during NEC	1	0.99 - 1	0.53
Maximum percentage of premature neutrophils at disease onset [%]	1	1 - 1.1	0.06
Maximum percentage of premature WBC at disease onset [%]	1	0.98 - 1	0.47
Base excess at disease presentation [mmol/L]	0.78	0.68 - 0.87	<0.001
Maximum base excess during NEC [mmol/L]	0.74	0.64 - 0.83	<0.001
Maximum PTT after disease onset	1.1	1 - 1.2	0.13
Maximum INR during NEC	7.3	1.4 - 76	0.056

Table V: Univariate regression - risk of severe NEC

Value	OR	CI95%	P
Birth weight [g]	1	1 - 1	0.0026
Birth weight percentile	1	0.99 - 1	0.83
Birth length [cm]	0.91	0.84 - 0.97	0.0057
Birth length percentile	1	0.99 - 1	0.76
Birth head circumference [cm]	0.85	0.76 - 0.94	0.0032
Birth head circumference percentile	0.99	0.97 - 1	0.14
Gestational age [weeks.days]	0.98	0.96 - 0.99	0.0014
Number of infants in this birth	0.6	0.3 - 1.1	0.12
Assisted delivery	1.1	0.43 - 3.3	0.81
Apgar 1 minute	0.81	0.69 - 0.95	0.011
Apgar 5 minutes	0.81	0.66 - 0.98	0.031
Apgar 10 minutes	0.76	0.6 - 0.94	0.014
Congenital heart disease	2.6	1.2 - 5.8	0.015
PDA	3.3	1.6 - 6.9	0.0012
Congenital malformations or syndroms	0.9	0.36 - 2.1	0.81
Age at diagnosis	1	0.97 - 1	0.93
Conservative treatment only	0.017	0.004 - 0.047	<0.001
NEC surgery	61	21 - 223	<0.001
More than one surgery due to NEC	15	6.4 - 36	<0.001
Male gender	1.3	0.66 - 2.7	0.44
Year of birth	1.1	0.96 - 1.2	0.21
Re-NEC	0.35	0.018 - 2	0.33
Umbilical artery pH	0.95	0.012 - 101	0.98
Umbilical venous pH	0.07	0 - 25	0.38

Table VI: Multivariate regression - risk of severe NEC

Value	OR	CI95	P
Survival: last known status	infinity	N/A	1
Birth weight [g]	0.883	N/A	1
Birth weight percentile	0.7087	N/A	1
Birth length [cm]	infinity	N/A	1
Birth length percentile	0.6127	N/A	1
Birth head circumference [cm]	0	N/A	1
Birth head circumference percentile	4.1948	N/A	1
Gestational age [weeks.days]	2.9501	N/A	1
Number of infants in this birth	0.0013	N/A	1
Assisted delivery	infinity	N/A	1
Apgar 1 minute	infinity	N/A	1
Apgar 5 minutes	0	N/A	1
Apgar 10 minutes	1e-04	N/A	1
Congenital heart disease	infinity	N/A	1
PDA	0	N/A	1
Congenital malformations or syndroms	0	N/A	1
Age at diagnosis	0.1738	N/A	1
Conservative treatment only	0	N/A	1
More than one surgery due to NEC	0	N/A	1
Male gender	infinity	N/A	1
Year of birth	0.0297	N/A	1
Re-NEC	0	N/A	1
Umbilical artery pH	infinity	N/A	1
Minimum Hb [g/l] <7 days before NEC onset	0.2923	N/A	1
Hb at disease presentation [g/l]	1.6543	N/A	1
Minimum Hb during NEC [g/l]	0.5405	N/A	1
Minimum WBC [G/L] <7 days before NEC onset	44.2766	N/A	1
Maximum WBC [G/L] <7 days before NEC onset	0.0088	N/A	1
WBC at disease presentation [G/l]	0.3705	N/A	1
Maximum WBC [G/l] during NEC	1.3859	N/A	1
Minimum PLT [G/l] <7 days before NEC onset	1.1211	N/A	1
Minimum PLT at disease presentation [G/l]	0.8327	N/A	1
Minimum PLT [G/l] during NEC	1.0583	N/A	1
Maximum total bilirubin <7 days before NEC onset [mcmol/l]	1.1107	N/A	1
Lactate at disease presentation [mmol/L]	0.4488	N/A	1
Maximum Lactate [mmol/l] during NEC	0.2884	N/A	1
CRP at disease presentation [mg/L]	1.1236	N/A	1
Maximum CRP [mg/l] during NEC	0.6618	N/A	1
Base excess at disease presentation [mmol/L]	0.5579	N/A	1
Maximum base excess during NEC [mmol/L]	1.0105	N/A	1

Table VII: Univariate regression - risk of severe NEC

Value	OR	CI95%	P
Minimum Hb [g/l] before NEC onset	0.99	0.97 - 1	0.09
Maximum Hb [g/l] before NEC onset	1	0.98 - 1	0.68
Minimum Hb [g/l] <7 days before NEC onset	0.99	0.98 - 1	0.22
Minimum Hb [g/l] 1-2 weeks before NEC onset	0.99	0.98 - 1	0.55
Hb at disease presentation [g/l]	0.99	0.97 - 1	0.019
Minimum Hb during NEC [g/l]	0.97	0.95 - 0.98	<0.001
Minimum WBC [G/L] before NEC onset	0.98	0.88 - 1.1	0.75
Maximum WBC [G/L] before NEC onset	1	0.95 - 1.1	0.92
Minimum WBC [G/L] <7 days before NEC onset	1	0.97 - 1.1	0.35
Maximum WBC [G/L] <7 days before NEC onset	1	0.98 - 1.1	0.22
WBC at disease presentation [G/l]	1	0.99 - 1.1	0.15
Maximum WBC [G/l] during NEC	1.1	1 - 1.1	0.0018
Minimum PLT [G/l] before NEC onset	1	0.99 - 1	0.25
Minimum PLT [G/l] <7 days before NEC onset	1	0.99 - 1	0.21
Minimum PLT at disease presentation [G/l]	0.99	0.99 - 1	0.0014
Minimum PLT [G/l] during NEC	0.99	0.98 - 0.99	<0.001
Maximum total bilirubin <7 days before NEC onset [mcmol/l]	1	1 - 1	0.41
Maximal lactate [mmol/l] before NEC onset	1.3	0.95 - 1.7	0.1
Maximal lactate [mmol/l] <7 days before NEC onset	1.4	1 - 2.2	0.062
Lactate at disease presentation [mmol/L]	1.5	1.3 - 1.9	<0.001
Maximum Lactate [mmol/l] during NEC	1.7	1.4 - 2.1	<0.001
Minimum pH before NEC onset	0.64	0.001 - 354	0.89
Minimum pH <7 days before NEC onset	0.0056	0 - 2	0.088
Maximum CRP before NEC onset	1	0.97 - 1	0.74
Maximum CRP [mg/l] <7 days before NEC onset	0.99	0.96 - 1	0.71
CRP at disease presentation [mg/L]	1	0.99 - 1	0.16
Maximum CRP [mg/l] during NEC	1	1 - 1	<0.001
Maximum percentage of premature neutrophils at disease onset [%]	1	1 - 1.1	0.0014
Maximum percentage of premature WBC at disease onset [%]	1	1 - 1.1	0.034
Base excess at disease presentation [mmol/L]	0.75	0.66 - 0.84	<0.001
Maximum base excess during NEC [mmol/L]	0.71	0.62 - 0.8	<0.001
Maximum PTT after disease onset	1	0.97 - 1	0.95
Maximum INR during NEC	23	2.9 - 312	0.0081

Table VIII: Predictors of severe NEC

Value	ROC-analysis						Univariate regression		
	AUC	cutoff	sensitivity	specificity	PPV	NPV	OR	CI95%	P
Birth length [cm]	0.64	39	0.6	0.7	0.44	0.82	3.5	1.7 - 7.5	<0.001
Birth head circumference [cm]	0.67	28	0.69	0.62	0.45	0.82	3.7	1.7 - 8.6	0.0013
Gestational age [weeks.days]	0.67	31.2	0.75	0.6	0.42	0.86	4.5	2.1 - 10	<0.001
Apgar 1 minute	0.64	5.5	0.64	0.57	0.36	0.81	2.4	1.2 - 5.1	0.019
Apgar 5 minutes	0.65	7.5	0.67	0.64	0.41	0.84	3.2	1.5 - 7	0.0033
Congenital heart disease	NA	NA	NA	NA	NA	NA	2.6	1.2 - 5.8	0.015
PDA	NA	NA	NA	NA	NA	NA	3.3	1.6 - 6.9	0.0012
Conservative treatment only	NA	NA	NA	NA	NA	NA	0.017	0.004 - 0.047	<0.001
NEC surgery	NA	NA	NA	NA	NA	NA	61	21 - 223	<0.001
More than one surgery due to NEC	NA	NA	NA	NA	NA	NA	15	6.4 - 36	<0.001
Minimum Hb during NEC [g/l]	0.71	110	0.68	0.66	0.43	0.85	2.8	1 - 7.5	0.042
Maximum WBC [G/l] during NEC	0.68	22	0.65	0.71	0.45	0.85	4	1.8 - 9.3	<0.001
Lactate at disease presentation [mmol/L]	0.75	2.6	0.67	0.74	0.47	0.86	3.7	1.7 - 8.3	0.0012
Maximum Lactate [mmol/l] during NEC	0.82	3.8	0.72	0.8	0.55	0.89	4.2	1.9 - 9.7	<0.001
Maximum percentage of premature neutrophils at disease onset [%]	0.72	34	0.65	0.73	0.44	0.87	2.9	1.2 - 7.4	0.025
Maximum INR during NEC	0.79	1.4	0.74	0.79	0.78	0.75	10	2.5 - 53	0.0022

Table IX: Multivariate regression - risk of death

Value	OR	CI95	P
Bell III	0	N/A	1
Birth weight [g]	0.9865	N/A	1
Birth weight percentile	0.3606	N/A	1
Birth length [cm]	infinity	N/A	1
Birth length percentile	0.5646	N/A	1
Birth head circumference [cm]	0	N/A	1
Birth head circumference percentile	3.5629	N/A	1
Gestational age [weeks.days]	0.4212	N/A	1
Number of infants in this birth	2.2644	N/A	1
Assisted delivery	1.4503	N/A	1
Apgar 1 minute	0.9566	N/A	1
Apgar 5 minutes	infinity	N/A	1
Apgar 10 minutes	0	N/A	1
Congenital heart disease	infinity	N/A	1
PDA	infinity	N/A	1
Congenital malformations or syndroms	0	N/A	1
Age at diagnosis	2.4191	N/A	1
Conservative treatment only	0	N/A	1
More than one surgery due to NEC	0	N/A	1
Male gender	0.7794	N/A	1
Year of birth	0.8336	N/A	1
Re-NEC	infinity	N/A	1
Umbilical artery pH	infinity	N/A	1
Minimum Hb [g/l] <7 days before NEC onset	0.7397	N/A	1
Hb at disease presentation [g/l]	1.514	N/A	1
Minimum Hb during NEC [g/l]	0.6897	N/A	1
Minimum WBC [G/L] <7 days before NEC onset	1.3417	N/A	1
Maximum WBC [G/L] <7 days before NEC onset	0.5367	N/A	1
WBC at disease presentation [G/l]	1.3708	N/A	1
Maximum WBC [G/l] during NEC	1.0823	N/A	1
Minimum PLT [G/l] <7 days before NEC onset	0.981	N/A	1
Minimum PLT at disease presentation [G/l]	0.9967	N/A	1
Minimum PLT [G/l] during NEC	0.9726	N/A	1
Maximum total bilirubin <7 days before NEC onset [mcmol/l]	1.046	N/A	1
Lactate at disease presentation [mmol/L]	2.2683	N/A	1
Maximum Lactate [mmol/l] during NEC	0.012	N/A	1
CRP at disease presentation [mg/L]	1.2617	N/A	1
Maximum CRP [mg/l] during NEC	0.8143	N/A	1
Base excess at disease presentation [mmol/L]	2.0216	N/A	1
Maximum base excess during NEC [mmol/L]	0.0096	N/A	1

Table X: Types of CHD in the patient cohort

Type	Total	Survivors	Non-Survivors	Bell III	Bell II
diseases with relevant reverse abdominal flow (A)	4	1	3	2	2
diseases with lower systolic output (B)	5	3	2	2	3
cyanotic heart diseases (C)	2	1	1	0	2
diseases with shunts (D)	21	18	3	10	11
pulmonary hypertension (E)	3	2	1	1	2
